# Effects of heat stress on endocrine, thermoregulatory, and lactation capacity in heat-tolerant and -sensitive dry cows

**DOI:** 10.3389/fvets.2024.1405263

**Published:** 2024-07-09

**Authors:** Xiaoyang Chen, Chenyang Li, Tingting Fang, Junhu Yao, Xianhong Gu

**Affiliations:** ^1^State Key Laboratory of Animal Nutrition and Feeding, Institute of Animal Science, Chinese Academy of Agricultural Sciences, Beijing, China; ^2^College of Animal Science and Technology, Northwest A&F University, Xianyang, Shanxi, China

**Keywords:** dry cows, heat stress, endocrine, thermoregulatory, lactation capacity

## Abstract

**Introduction:**

Increasing global temperatures present a significant challenge to livestock production. The dry period is an important stage in the production cycle of cow, and environmental heat stress (HS) during this period can have adverse effects on the subsequent lactation performance. In this study, we aimed to investigate the effects of HS on endocrine, thermoregulatory, and lactation parameters in heat-tolerant dry cows (HTDC) and heat-sensitive dry cows (HSDC).

**Methods:**

We measured the respiratory rate (RR), body temperature (BT), and temperature-humidity index (THI) in 66 dry cows during HS. The slopes of RR and BT to THI were determined through analysis of measurements and dry cows background information using a mixed-effects model. Subsequently, the heat tolerance or sensitivity of dry cows was assessed using clustering method (HTDC = 19 and HSDC = 47).

**Results:**

Compared with that of HSDC, the RR of HTDC significantly increased after exposure to HS (*p* < 0.05). The average reduction in milk yield from new lactation to the previous lactation was significantly lower in HTDC compared to HSDC (*p* < 0.05). Plasma cortisol and non-esterified fatty acid levels were significantly lower in HTDC compared to HSDC (*p* < 0.05), while plasma triiodothyronine (*p* = 0.07) and growth hormone (*p* = 0.08) levels tended to be higher in HTDC relative to HSDC.

**Discussion:**

HTDC can more effectively alleviate the impacts of HS through their superior thermoregulation and metabolism, thereby ensuring optimal postpartum lactation performance.

## 1 Introduction

The 2019 global climate report emphasized the acceleration of the global warming trend, forecasting a possible record-setting scenario in the upcoming five years (1). The World Meteorological Organization has cautioned that the escalating global temperatures present a significant challenge to livestock production, with temperature rises of 3 to 5°C anticipated during this century (2). Climatologists have extensively researched climate parameters in Shandong Province, China, and identified a general pattern of rising temperatures and decreasing precipitation in the region (3). Climatic conditions pose a primary constraint on animal welfare, physiological development and production. Climate change-induced heat stress (HS) has far-reaching consequences for all living organisms. HS has significant effects on cattle health, reproduction, growth, and welfare (4).

The dry period is an important stage in the production cycle of cow; however, due to its nonproductive nature, people often neglect active management during this stage (5). The negative effects of HS in dry cows on the profitability of dairy farms (6) are similar to those during lactation (7). Compared to lactating cows, dry cows produce less metabolic heat (8, 9). Despite these advantages, environmental HS during the dry period negatively affects subsequent lactation performance (6, 10–13). In addition, dry period exposure to stressors could be detrimental to fetal and mammary growth in heifers (14). The negative effects of HS exposure in dry cows have been studied extensively but heat tolerance of dry cows to HS is unknown (15). Therefore, it is important to understand the heat tolerance and heat sensitivity of cows during dry periods to determine the effects of HS on different tolerance cow on the same farm.

Behavioral and physiological adaptations occur under HS to maintain basic body functions. Many measures have been proposed as criteria for identifying heat-tolerant animals, including heart rate, sweating rate, respiration rate (RR), and body temperature (BT) (16). Pinto (17) reported that the RR of cows increased with increasing THI. Gaughan (18) demonstrated that the influence of the ambient temperature on RR. Synthesizing results of these studies, we propose that the slopes of RR and BT to THI, determined by analyzing RR, BT, and cows background information using a mixed-effects model, be used as selection criterion for determining heat tolerance (19). Carabaño (20) reported a similar conclusion, they assessed the heat tolerance of cows by the slope of production traits and somatic cell scores relative to ambient temperature. In our study with lactating cows (19), we selected heat-tolerant and heat-sensitive cows by using mixed models and studied the effect of HS on these two types of cows. Our study indicated that heat-tolerant cows generally showed steeper slope in RR and BT, and conversely, the decay in milk yield was lower than heat-sensitive cows. Furthermore, our study identified RR and BT as the most critical indices in the screening model for heat tolerance and heat sensitivity in lactating cows. Therefore, we believe that we can assess heat tolerance and heat sensitivity in dry cows using this model. In summary, simultaneously assessing RR and BT enables the evaluation of heat tolerance and sensitivity in dry cows, leading to mitigating the effects of HS in dry cows through better management.

Adaptation to HS requires the endocrine, immune systems, and cardiorespiratory to operate in concert (21). Neuroendocrine regulation is a primary adaptive response exhibited by animals under extreme stress conditions (22). HS affects endocrinology in dairy cows, resulting in the release of thyroxine (T4), cortisol (COR), and growth hormone (GH) (23). COR is closely related to behavior and neuroendocrinology during HS (24). Therefore, COR is the most important indicator for assessing stress levels. Cattle farms use blood levels of heat shock protein-70 (HSP-70) as a reliable indicator for identifying HS (25). Under HS conditions, heat shock protein-90 (HSP-90) increase cell survivability and regulate BT (26). In summary, these blood indicators can be measured to validate the success of heat tolerance and heat sensitivity screening in dry cows, and to delve deeply into the endocrine differences between heat-tolerant dry cows (HTDC) and heat-sensitive dry cows (HSDC).

We hypothesized that HTDC would thermoregulate more effectively and reduce the impact of HS in the next lactation milk yield compared with HSDC. Herein, our study measured the RR and BT of dry cows along and the THI during measurement period. We identified HTDC and HSDC based on the slope of RR and BT relative to THI. Then, we investigated the effects of HS abatement on dry dairy cows’ thermoregulatory responses and lactation performance in their next lactation. As far as we are aware, our study is the first to describe heat tolerance and heat sensitivity selection for dry cows. We aimed to show the alterations in endocrine, thermoregulatory, and lactation capacity in HTDC and HSDC under HS conditions, and provide a reliable foundation for devising cost-effective HS mitigation options for dry cows.

## 2 Materials and methods

### 2.1 Experimental design and animal management

The experiment was carried out at an intensive organic dairy farm located on the east coast of Shandong, China, with geographical coordinates of 34° 50′ 37″ N, 115° 26′ 11″ E, and an altitude of 52 m. The study was conducted in a specialized barn designed for dry cows, situated within an organic, intensive dairy farm that relies solely on self-produced, antibiotic-free feed. The barn utilized for the research, which was specifically designed for dry cows, was divided into two sections: an indoor space measuring 15 m × 90 m and an outdoor area measuring 15 m × 45 m. The barn was equipped with a concrete floor covered in clean, dry grass bedding and lacked fixed beds. A double-pitched roof covers the entire indoor space, shielding the cows from direct sunlight. Electronic fans were installed in the indoor sections of the barn, positioned 6 m apart in the resting area and 12 m along the feeding line. Water sprinklers, placed every 2 m along the feeding line for cooling purposes, activated when the room temperature reached 20°C. Dry dairy cows were provided with a total mixed ration from 8:00 h to 8:30 h, 14:00 h to 14:30 h, and 19:00 h to 19:30 h each day, and all cows were watered freely throughout the study period. Dietary ingredients and nutritional content were provided by the intensive organic dairy farm (Table 1). The experienced veterinarians conducted daily examinations of the cows from 8:00 h to 9:00 h.

**Table 1:** Ingredients and nutrient composition of experimental diets.

Item	Value
Ingredients	Content, %
Whole corn silage	48.33
Alfalfa	10.39
Pressed corn	9.43
Soybean meal	8.70
Mineral and vitamin mix ^2^	3.70
Cottonseed	2.90
Oat hay	2.42
Beet pulp	2.42
Rapeseed meal	1.69
Extruded soybean	1.33
DDGS 1	0.72
Dandelion	0.48
Nutrient composition	
DM, % of wet TMR	62.40
NDF	35.75
ADF	18.20
CP	17.06
NEL/(MJ/kg)	6.11
EE	3.32

Dry matter (DM), Crude protein (CP), Ether extract (EE), Neutral detergent fiber (NDF), Acid detergent fiber (ADF), Total Mixed Ration (TMR), Net energy for lactation (NEL), Distillers dried grains with solubles (DDGS). ^1^DDGS, Distillers Dried Grains with Solubles. ^2^Contained the following per kg of diets: VA 170000 IU, VD 8000 IU, VE 19000 IU, Ca 160 g, P 50 g, Fe 800 mg, Cu 680 mg, Mn 3,500 mg, Zn7500 mg, Se 80 mg, I 400 mg, Co 38 mg.

This study screened healthy dry cows that had calved at least once and recently entered the dry period (~ 40 days before expected calving date), with a normal body condition score (BCS; between 3–4) (27). The BCS is evaluated by a veterinarian with expertise in dairy farming and the scoring chart was adopted from Edmonson (27). Sixty-six dry-milking Holstein-Friesian cows fulfilled the inclusion criteria. The mean ± standard deviation parity, BCS, and gestation time of the selected cows were 2.91 ± 0.78, 3.23 ± 0.35 and 244.11 ± 3.01 days, respectively. Information about the cows, including daily milk yield (covering two complete lactation cycles before and after the dry period), parity, days in milk, and age, was extracted from the milking systems (Afimilk, Kibbutz Afikim, Israel). All the cows were observed over a 7 days period, and samples were collected from these cows. Then we used a clustering method to identify dry cows tolerant or sensitive to HS. This identification was based on the slope of physiological indicator (RR and BT) in response to the THI during HS, then after cluster analysis, cows exhibiting higher RR and BT slopes were categorized into the heat-tolerant group (22). Conversely, the heat-sensitive group was identified for those dry cows with lower slopes. The entire study commenced on the tenth day of the dry period and extended until the conclusion of the postpartum period (July 10 to August 20, 2022), except for date collection for milk yield performance.

### 2.2 Environmental, physiological, and gestation indicator measurements

**Figure 1:**
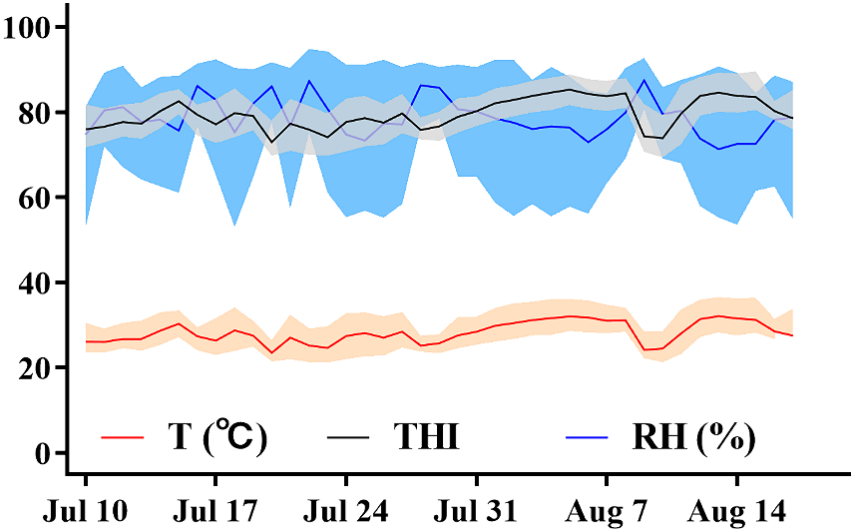
Daily minimum, maximum, and mean ambient temperature (°C), relative humidity (%), and THI during the environmental HS exposure period from July 10 to August 20, 2022, at the experimental cow farm in Shandong.

Environmental parameters were assessed near the cows and within the barn using both hand-held and stationary methods. The Kestrel 5,400 Heat Stress Trackers (Nielsen-Kellerman, Boothwyn, PA, USA) were used for these measurements. For the hand-held model, environmental parameters were measured 1 m away from the cow during physiological indicator measurements. For the stationary model, measurements were taken every 15 min at a fixed position in the middle of the barn using the Kestrel 5,400 Heat Stress Tracker. Both hand-held and stationary indices included ambient temperature (Ta) and relative humidity (RH). The hand-held index represents Ta and RH measured in the cows’ vicinity during the assessment of physiological indices in dry cows, while the stationary index represents environment Ta and RH measured in the barn. The hand-held model measures environmental parameters to select cows’ heat tolerance, while the stationary model records environmental parameters within the barn. Figure 1 describes the daily minimum, maximum, and mean ambient RH, Ta, and THI measured throughout the study. The THI was calculated using the formula provided by the National Research Council (28):


$$THI=\left(1.8\times Ta+32\right)-\left(0.55-0.0055\times RH\right)\times \left(1.8\times Ta-26\right)\text{.}$$

RR and BT were used as physiological indicators. RR was assessed by two trained postgraduates with a high level of agreement (intragroup correlation coefficient: 0.91), and was achieved by timing 15 flanking movements and converting them to breaths per minute (bpm) (19). BT was evaluated by IRT using a portable thermal imaging infrared camera (Fluke TiS60+ Thermal Imaging Camera, Fluke, Everett, Washington, USA) equipped with 320 × 240 pixel detector and adjusted to thermal sensitivity <45 mK (< 0.045°C at the ambient temperature of 30°C) and temperature range from −20 to 400°C, in the manual focus option. The emissivity was set to 0.98, and the distance between the cow and the camera was approximately 1.5 m, in accordance with previous studies (29). We used eye temperature as a measure of BT, as confirmed in our previous study (30). Physiological indicator measurements were conducted twice daily on each of the 7 test days, with one measurement at 08:00 h to 09:00 h and the other at 14:00 h to 15:00 h in each period. Each cow was observed twice for every measurement, once in a standing position and once while lying down. During each measurement, the position (e.g., feeding area, resting area, aisle area, and fan area) and behavior of the test cow (e.g., drinking, feeding, standing, and lying) were recorded for subsequent grouping in the experimental model.

When the dry cows had finished parturition, we recorded the gestation indicator (the duration of gestation, birth weight of the calf, delivery score, and colostrum quality). Delivery scores relate to the difficulty of the heifer’s delivery (unassisted: 1, easy pull: 2, and hard pull, mechanical extraction, cesarean section: 3) (31). We used the optical Brix refractometer for rapid on-farm assessment to determine colostrum quality (Manual Refractometer MHRB-40 ATC, Mueller Optronic, Erfurt, Germany).

### 2.3 Blood collection and processing

At the end of the physiological indicator measurement period, we collected 10 mL blood samples from the coccygeal vessels of 22 dry cows (10 and 12 dry cows randomly selected from the HTDC and HSDC). The collection took place from 06:00 h to 06:30 h in the morning and was stored in vacutainers (BD vacutainers EDTA tube, Fisher Scientific, Waltham, MA, USA). The blood samples were centrifuged at 3000 × g for 15 min at 4°C to obtain plasma, which was stored in liquid nitrogen. Insulin (INS), thyroxine (T4), growth hormone (GH), triiodothyronine (T3), cortisol (COR), and prolactin (PRL) were measured using a BFM-96 multitube radioimmunoassay analyzer (Hefei, China). Blood glucose (GLU) levels were measured using an AU480 auto-analyzer (Olympus Co., Tokyo, Japan). Heat shock protein-70 (HSP-70), heat shock protein-90 (HSP-90), 5-hydroxytryptophan (5-HT), interleukin-6 (IL-6), and non-esterified fatty acids (NEFA) were measured by ELISA assay according to the manufacturer’s instructions. Colorimetric data for all of the above measurements were measured using THERMO Multiskan Ascent (Waltham, MA, USA).

### 2.4 Statistical analysis

This study was conducted using SAS for all the statistical analyses (version 9.4, 2019, SAS Institute Inc., Cary, NC, USA) (32). This study used PROC MIXED to fit a mixed-effects model that uses data obtained during HS to quantify individual differences in dry cows’ heat tolerance and sensitivity. HTDC and HSDC were obtained from a hierarchical cluster analysis based on the Ward method using PROC CLUSTER after separate slopes for RR and BT of the THI responses were derived from the mixed-effects model (19). Levene’s test was employed to examine the data, and residual normality was assessed using the Shapiro–Wilk statistic through the UNIVARIATE procedure. The raw data were transformed, as necessary, to ensure homogeneity of variance, and subsequently, back-transformed for visual representation. Thermoregulatory responses analyzed by using generalized linear mixed models using PROC MIXED. The model incorporated fixed effects of two groups, time (day, as a repeated measure). The random effect included the animal identification number nested within the two groups and all possible interactions (zone and behavior). The model for analyzing weekly thermoregulatory responses included the THI at the time of sampling as a covariate.

PROC GLM was used to conduct a ANCOVA test to identify significant differences in calf birth weight, duration of gestation, delivery score, colostrum quality, milk yield, and plasma indicators between HTDC and HSDC, included BCS, parity, and age as covariates. All data are presented as least squares means ± standard deviations (SD). Significance and tendency were declared if *p* ≤ 0.05 and 0.05 < *p* < 0.10, respectively.

## 3 Results

**Figure 2:**
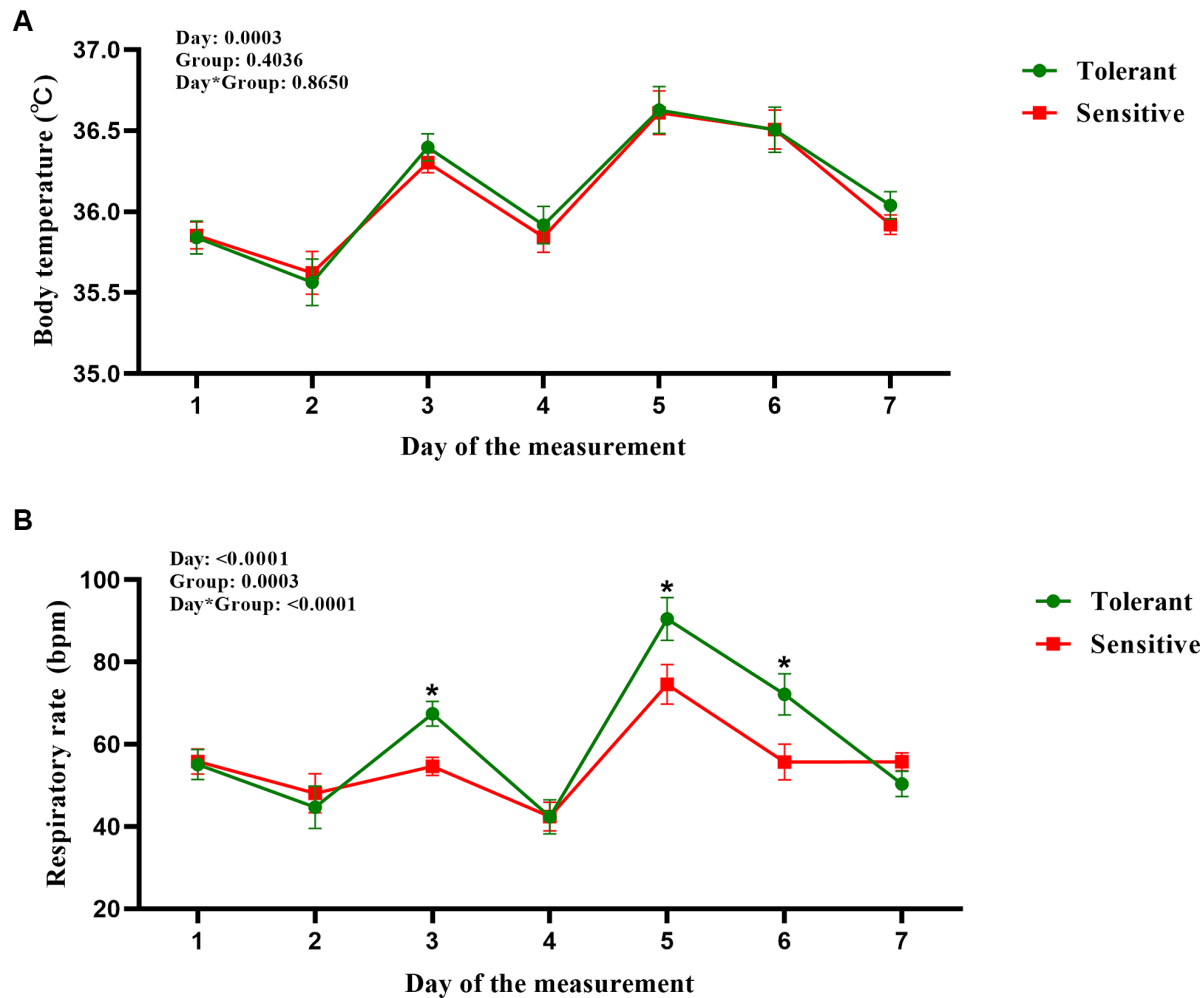
Body temperature **(A)** and respiration rate [**(B)**, bpm = breaths per minute] measurements from heat-tolerant or heat-sensitive heifers during the measurement 7 day. The horizontal coordinates (day of measurements) 1 to 7 corresponded to cows at −43 to −50 days relative to the expected date of parturition. Tolerant is shown in green (*n* = 19) and Sensitive is shown in red (*n* = 47). Data are graphed using the LSMean ± SD of the interaction (group by day). * indicates significance (*p* < 0.05).

Utilizing mixed-effect models and cluster analysis on RR and BT from 66 cows, we identified HTDC and HSDC (Table 2); the slopes of RR and BT to THI for HTDC (*n* = 19) were 2.55 ± 0.27 and 0.17 ± 0.05, while in the HSDC (*n* = 47), the corresponding slopes were 1.7 ± 0.38 and 0.16 ± 0.03. During the 7 days of our measurement, the difference between the two groups in terms of BT was not significant (*p* > 0.05; Figure 2A), with only a significant effect of day (*p* = 0.0003) but no group by day interaction (*p* = 0.8650). In contrast, the HTDC exhibited a significantly higher RR compared to the HSDC (55.11 ± 14.11 vs. 61.26 ± 19.23 bmp, *p* = 0.0003; Figure 2B). There was a significant effect of both the day and group by day interactions (all *p* < 0.0001), showing RR was significantly higher in HTDC compared to HSDC on days 3, 5, and 6 of measurement. Table 3 reveals that there were no significant differences in the duration of gestation, parturition score, colostrum quality, and birth weight of calves born to cows in the HTDC and HSDC post-parturition (*p* > 0.05). All dry cows were in healthy condition throughout the study period.

**Table 2:** Clustering results based on the individual slopes of respiration rate (RR) and body temperature (BT) in dry cows, with respect to THI.

Items	Heat-tolerant		Heat-sensitive	
	Mean	Standard deviation	Mean	Standard deviation
*RR* (bpm/THI)	2.55	0.27	1.70	0.38
*BT* (°C/THI)	0.17	0.05	0.16	0.03

Heat-tolerant dry cows: *n* = 19, heat-sensitive dry cows: *n* = 47.

**Table 3:** Differences on birth weight of calves, duration of gestation, delivery score, and colostrum quality among heat-tolerant dry cows (*n* = 19) and heat-sensitive dry cows (*n* = 47).

Items	Groups		*p*-value
	Heat-tolerant	Heat-sensitive	
Birth weight of calves (Kg)	37.68 ± 6.07	37.57 ± 5.01	0.99
Duration of gestation (Day)	273.95 ± 3.76	273.77 ± 5.59	0.88
Delivery score	1.05 ± 0.23	1.06 ± 0.25	0.87
Colostrum quality (%)	26.53 ± 1.13	26.47 ± 0.93	0.83

**Figure 3:**
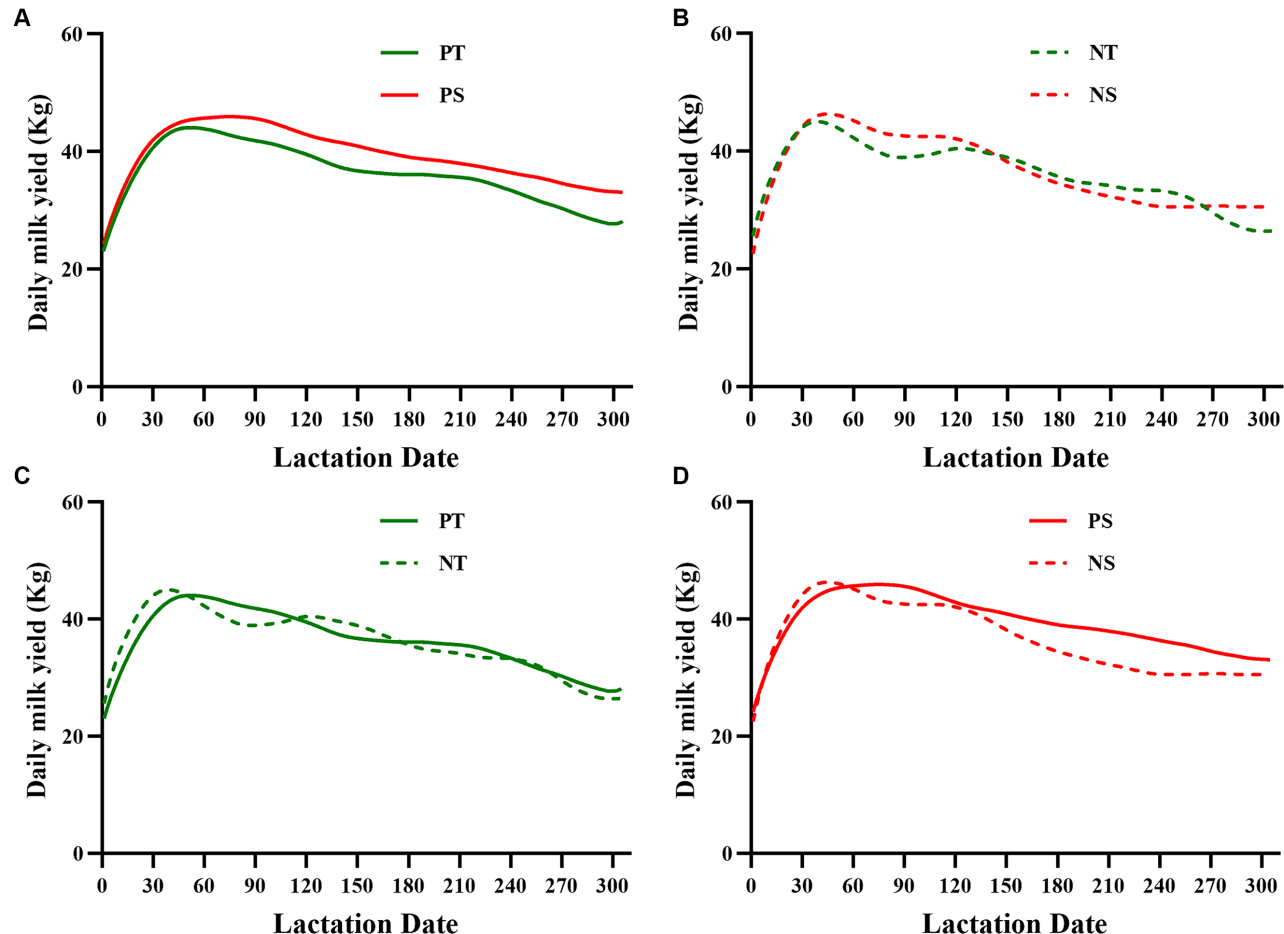
The previous and new lactation curves (305 d) of both heat-tolerant (T) and -sensitive (S) groups were compared **(A,B)**. Additionally, the new lactation curves were compared to the previous ones within each group **(C,D)**. T shown in green (*n* = 12) and S shown in red (*n* = 26). PT, Previous lactation curve (Heat-tolerant group); PS, Previous lactation curve (Heat-sensitive group); NT, New lactation curve (Heat-tolerant group); NS, New lactation curve (Heat-sensitive group).

A total of 38 test dry cows had milk yield data collected for two full lactation cycles (305 days, including milk yield data for both pre-and post-dry lactation periods) due to farm equipment and herd adjustments (Table 4). HTDC and HSDC, with 12 and 26 cows having complete lactation data, respectively, showed no significant difference in average milk yield between the previous and new lactations (Table 4, *p* > 0.05). However, the reduction value in average milk yield from the new lactation to the previous lactation was significantly higher in HSDC than in HTDC (Table 4, *p* < 0.05). Prior to exposure to HS during the dry period, the HSDC had a higher lactation curve than HTDC (Figure 3A). Then it was found by Figures 3A,B that the difference in lactation curves between HTDC and HSDC became narrower after exposure to HS during the dry period. Additionally, in certain periods, HTDC surpassed HSDC (Figure 3B). Minimal variation and only slight fluctuations were observed in the previous and new lactation curves of HTDC after exposure to HS (Figure 3C), whereas the previous and new lactation curves of HSDC exhibited larger variability and fluctuation (Figure 3D).

**Table 4:** Differences on milk yield (305 d) among heat-tolerant dry cows (*n* = 12) and heat-sensitive dry cows (*n* = 26).

Items	Groups		*p*-value
	Heat-tolerant	Heat-sensitive	
Previous milk yield (Kg/d)	36.67 ± 6.11	39.81 ± 6.76	0.18
New milk yield (Kg/d)	36.56 ± 7.65	36.99 ± 7.62	0.87
Reduction value (Kg/d)	−0.11 ± 3.51	−2.81 ± 3.75	0.04

**Figure 4:**
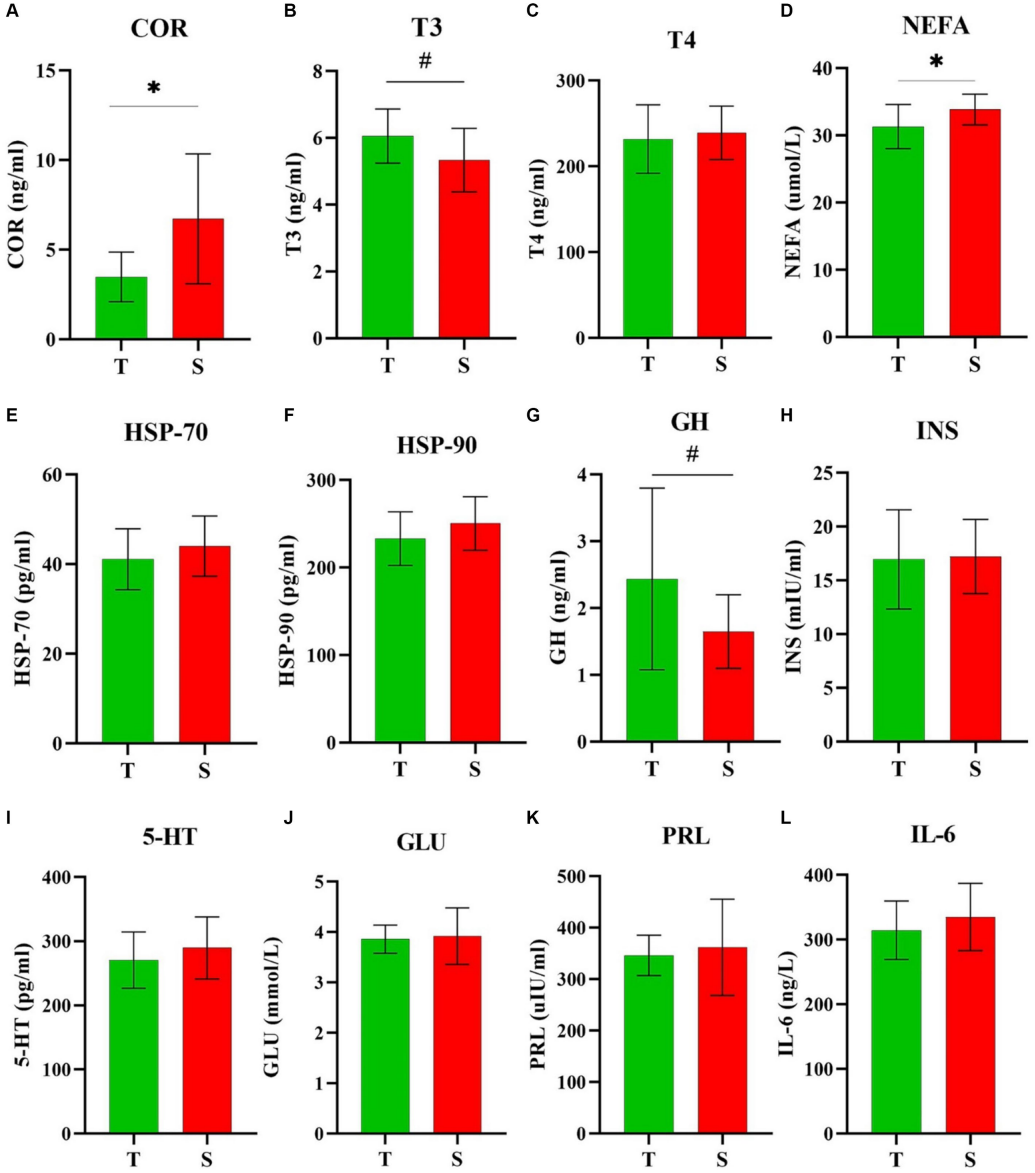
Comparison of plasma biochemical analysis. Plasma levels of **(A)** COR, **(B)** T3, **(C)** T4, **(D)** NEFA, **(E)** HSP-70, **(F)** HSP-90, **(G)** GH, **(H)** INS, **(I)** 5-HT, **(J)** GLU, **(K)** PRL, and **(L)** IL-6 among heat-tolerant (T) and -sensitive (S) groups. *and ^#^ indicates significance (*P* < 0.05) and tendence (0.10 ≥ *P* > 0.05), respectively. T shown in green (*n* = 10) and S shown in red (*n* = 12).

The plasma indicator results obtained through the analysis of collected blood are presented in Figure 4. HTDC exhibited lower plasma COR (Figure 4A) and NEFA (Figure 4D) levels compared to HSDC (*p* < 0.05). HTDC tended to exhibit higher plasma T3 (Figure 4B) and GH (Figure 4G) levels compared to HSDC (*p* = 0.07 and *p* = 0.08), respectively. However, these were no significant difference between HTDC and HSDC in other plasma indicators shown in Figure 4 (*p* > 0.05).

## 4 Discussion

Essentially, heat-resistant animals can maintain a constant temperature in hot environments (33). The ability of an animal to maintain a stable temperature under HS conditions depends on the ability to balance heat production and dissipation (34). In our study, we categorized dry cows into two distinct groups by estimating the slopes of RR and BT in relation to the THI using a mixed-effects model. This categorization was achieved through hierarchical cluster analysis after standardizing the individual slopes. Consistent with our previous study (19), the group characterized by fewer numbers and relatively steeper slopes after clustering was identified as heat-tolerant cows, while the opposite was heat-sensitive cows. The present study expands upon screening methods for selecting heat tolerance in dry cows under thermal environmental conditions. It also evaluated the effects of heat stress on endocrine, thermoregulatory, and lactation capacity. Dry cows were selected for heat tolerance and sensitivity in a noninvasive manner in a thermal environment to assist in analyzing their thermal conditions and understanding their adaptation to the thermal environment. In present study, all dry cows were housed in the same barn and experienced similar levels of THI, which were consistently above 68 for the duration of the experiment. In the previous study, rectal temperature and RR were lower in cooled cows compared to cows exposed to HS (7, 35). However many studies have consistently demonstrated that Jersey cows have a significantly higher RR than Holstein cows due to their superior heat dissipation ability (36). Cattle use skin temperature to increase heat loss due to convection and radiation, and increase RR to increase respiratory evaporative heat loss to cope with a heat environment (37). Cattle may increase RR during cooler nighttime periods to enhance heat dissipation (18, 38). Dairy cows respond to HS by reducing feed intake, decreasing productivity, and increasing RR (39). The disparity in outcomes across the aforementioned studies may stem from differences in study methods. In the HS relief studies, the RR of cows subjected to cooling treatment was lower than that of the HS group. Conversely, in studies of heat tolerance in dairy cows experienced HS just utilized its own heat-resistant ability, they naturally acclimated to the heat environment by increasing their RR. Our study confirmed the conclusion that HTDC exhibit a steeper RR slope when responding to HS by elevating their RR more promptly to enhance heat dissipation. At the same time, skin is a crucial heat exchange pathway, with BT regulated by blood flow between the body core and skin (40). Collier (41) showed that BT was significantly higher in HS cows than in non-HS cows. Many studies have shown that under HS, heat-tolerant cows have lower RR, BT, or better sweating rates and reproductive performance than heat-sensitive cows (16). In the previous study, BT of heat tolerant cows were not significantly different from heat sensitive cows during lactation (19). The same results were obtained in the present study, with no difference in BT between HTDC and HSDC after suffering HS. We attribute this result to the availability of cooling opportunities during the study period, which resulted in relatively minor changes in BT observed in all healthy cows exposed to HS, leading to no differences between HTDC and HSDC. In addition, the inadequate frequency of BT measurements may also contribute to this outcome.

Previous studies indicated that HS reduced gestation length compared with that of cooled dry cows (11). Newborns dams exposed to HS in late gestation had lower birth weights than cooler dams (42). The study by Fabris (7) found that cooled cows had heavier calves at birth than HS cows, which was attributed to the longer gestation period cooled cows have compared to HS cows during the dry period. However, our study yielded different conclusions compared to previous studies. This disparity may be attributed to the cooling measures implemented during our study, which mitigated these issues in the heifers. This may also explain why there was no difference between HTDC and HSDC delivery scores and colostrum quality in our study. Previous studies have shown that cooling applied throughout the dry period during HS leads to increased milk yield in the next lactation period (43). Cows cooled throughout the dry period produced more milk yield in the next lactations than cows in shade only (44). Additionally, our study showed a similar conclusion. Specifically, the average decrease in milk yield among HTDC was significantly lower than HSDC under identical environmental conditions. Furthermore, HTDC exhibited less variability and fluctuations in the lactation curve following exposure to HS. The previous studies appeared to explain the results, cows increase milk yield in the next lactation through cellular renewal of the mammary gland during the dry period, mammary redevelopment during the dry period is critical for maximal subsequent production (45). Compared with HSDC exposed to HS, the HTDC identified in our study seemed to exhibit superior adaptation to HS and a greater capacity for mammary redevelopment during this period.

Under HS conditions, cows use thermoregulatory mechanisms to adapt to HS, mechanisms include reduced metabolic rate, altered blood hormone concentrations, and increased RR and BT (7). HS triggers a response in cows characterized by the neurologic and glandular secretion of hormones, resulting in endocrine alterations, notably changes in glucocorticoid, GH, T4, and PRL concentrations (46). The modulation of hormonal profiles during HS periods depends on various factors, including the duration and intensity of exposure HS (47, 48). HS increases COR and PRL levels in cows (21). COR is the primary glucocorticoid that plays a key role in most mammals, including cattle (49). Blood COR levels are often used as a reliable biomarker for stress, indicating animals’ responses to different stress levels (50). Our study yielded HTDC had lower COR levels than HSDC, suggesting that HTDC had lower HS levels compared to HSDC. And elevated COR levels affect the synthesis of heat shock proteins (HSPs) (51). HSPs, such as HSP-70, play a crucial role in animal metabolism, and an increase in their expression can lead to increased GLU levels, and activate the immune system (52). Min (53) found that serum HSP-70 and HSP-90 levels increased significantly during HS. Du (54) reported that the blood IL-6 have decreased in the heat-stress healing group. Additionally, 5-HT plays a key role in the central thermoregulatory system (55). There were no differences in plasma HSP-70, HSP-90, GLU, IL-6, PRL, and 5-HT between HTDC and HSDC in this study. It might be attributed to the fact that all dry cows were exposed to HS during the study period and the number of blood samples collected was in small quantities. Thyroid hormones (T3 and T4), GH, COR, and mineralocorticoids were the main hormones associated with HS adaptation in dairy cows (56). HS leads to decreased thyroid hormone levels in cows (47). Previous studies have shown that the plasma GH was significantly reduced in Jersey cows affected by HS (57) but increased plasma NEFA levels in cows (58). These conclusions explained why plasma T3, GH, NEFA and COR levels were different between HTDC and HSDC exposed to HS. COR levels suggested that HTDC experienced less stress compared to HSDC, while T3, GH, and NEFA levels indicated that HTDC exhibited a stronger thermoregulatory ability and metabolic capacity than HSDC. In all, our study effectively identified HTDC and HSDC, offering insights for precise and intelligent feeding management of dry cows during summer. Further research is necessary to confirm this conclusion, which may involve increasing the sample size and breeds of cows, etc.

## 5 Conclusion

In summary, our study used mixed-effects model and clustering analysis to categorize dry cows into two groups based on their heat tolerance and sensitive during HS period. HTDC experience lower stress levels compared to HSDC when exposed to HS under the same environmental conditions. Additionally, they exhibit superior thermoregulation and metabolism. HTDC can mitigate the effects of HS more rapidly through their thermoregulatory system, thereby ensuring optimal post-calving lactation performance. In this study, HTDC and HSDC could be distinguished and separated to facilitate precise feeding management to optimize the economic benefits of summer farming.

## Data availability statement

The original contributions presented in the study are included in the article/supplementary material, further inquiries can be directed to the corresponding author.

## Ethics statement

All procedures were approved by the Experimental Animal Welfare Ethical Committee, Institute of Animal Science, Chinese Academy of Agricultural Sciences (approval number IAS2022-112). The studies were conducted in accordance with the local legislation and institutional requirements. Written informed consent was obtained from the owners for the participation of their animals in this study.

## Author contributions

XC: Data curation, Investigation, Methodology, Software, Supervision, Validation, Visualization, Writing – original draft, Writing – review & editing. CL: Investigation, Project administration, Supervision, Validation, Writing – review & editing. TF: Writing – review & editing. JY: Methodology, Supervision, Writing – review & editing. XG: Conceptualization, Funding acquisition, Investigation, Project administration, Resources, Software, Supervision, Writing – original draft, Writing – review & editing.
