# Corrigendum: Effects of heat stress on endocrine, thermoregulatory, and lactation capacity in heat-tolerant and -sensitive dry cows

**DOI:** 10.3389/fvets.2024.1463893

**Published:** 2024-07-30

**Authors:** Xiaoyang Chen, Chenyang Li, Tingting Fang, Junhu Yao, Xianhong Gu

**Affiliations:** ^1^State Key Laboratory of Animal Nutrition and Feeding, Institute of Animal Science, Chinese Academy of Agricultural Sciences, Beijing, China; ^2^College of Animal Science and Technology, Northwest A&F University, Xianyang, Shanxi, China

**Keywords:** dry cows, heat stress, endocrine, thermoregulatory, lactation capacity

In the published article, there was an error in Figure 4 as published. In the figure caption, “n” for “T” (in green), and “S” (in red), were incorrectly defined as 19 and 47, respectively. The corrected Figure 4 and its revised caption can be found below.

**Figure 4:**
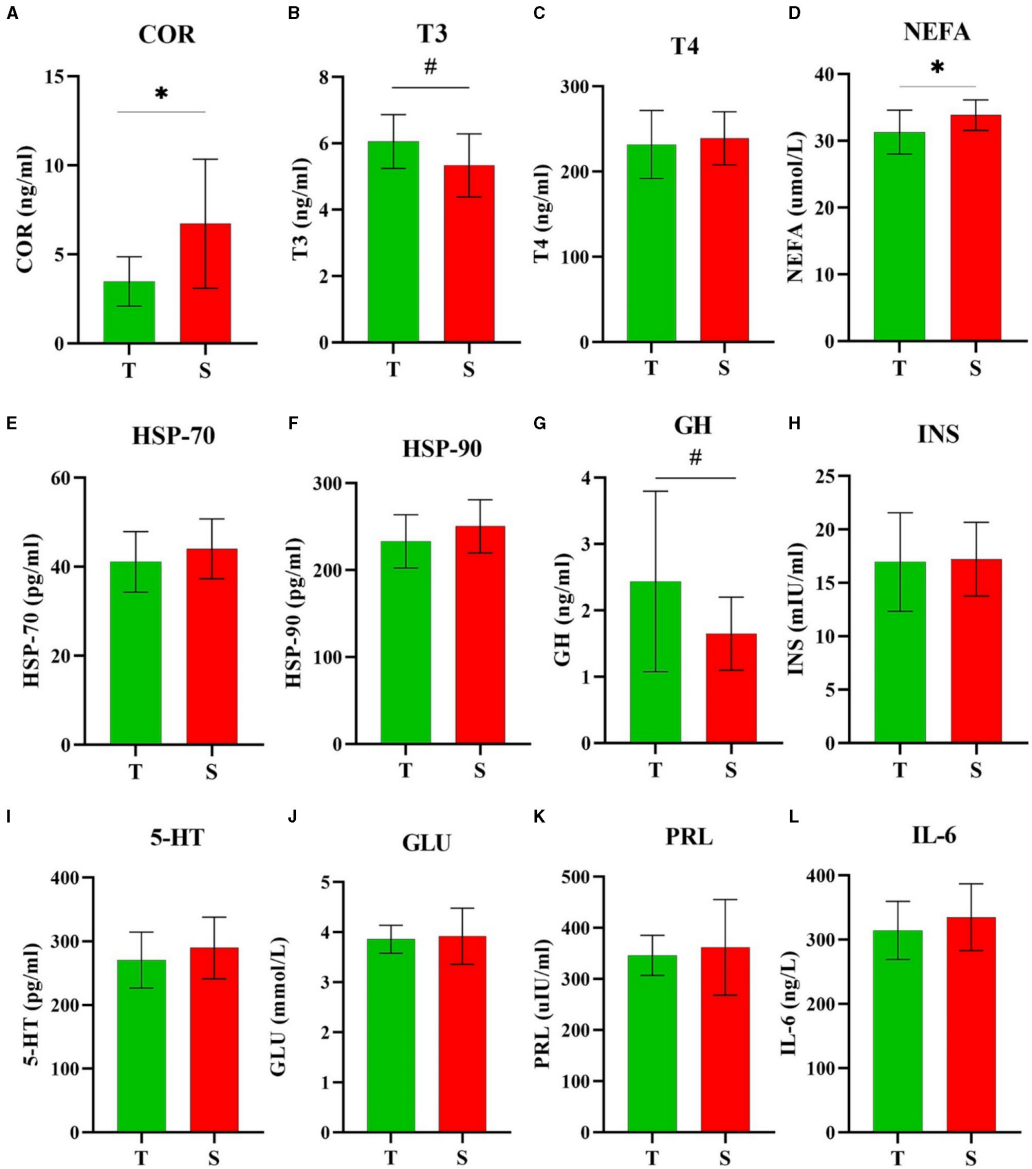
Comparison of plasma biochemical analysis. Plasma levels of **(A)** COR, **(B)** T3, **(C)** T4, **(D)** NEFA, **(E)** HSP-70, **(F)** HSP-90, **(G)** GH, **(H)** INS, **(I)** 5-HT, **(J)** GLU, **(K)** PRL, and **(L)** IL-6 among heat-tolerant (T) and -sensitive (S) groups. ^*^and # indicates significance (*P* < 0.05) and tendence (0.10 ≥ *P* > 0.05), respectively. T shown in green (*n* = 10) and S shown in red (*n* = 12).

The authors apologize for this error and state that this does not change the scientific conclusions of the article in any way. The original article has been updated.

